# Peritoneal adenocarcinoma diagnosed by endoscopic ultrasound-guided through-the-needle biopsy

**DOI:** 10.1055/a-2320-2098

**Published:** 2024-06-05

**Authors:** Zhenyun Gong, Guilian Cheng, Wei Wu, Duanmin Hu

**Affiliations:** 1 105860 Gastroenterology, Second Affiliated Hospital of Soochow University, Suzhou, China

The diagnosis of peritoneal carcinomatosis accompanied by ascites is challenging, particularly when the primary site remains unidentified. While cytological examination of ascites is commonly advocated, the diagnostic sensitivity is approximately 60% 1 . The remaining 40% of patients may require more invasive procedures, such as laparoscopy or laparotomy. In recent years, endoscopic ultrasound (EUS) has extended its reach to peritoneal carcinomatosis, offering enhanced visualization and a minimally invasive approach 2 3 . In the case presented here, we utilized an innovative technique, EUS-guided through-the-needle (EUS-TTN) biopsy, to facilitate the pathological diagnosis of peritoneal carcinomatosis.

A 77-year-old woman was referred to our hospital for abdominal distension. Computed tomography demonstrated abnormal peritoneal thickening alongside significant ascites accumulation without detecting any space-occupying lesion ( Fig. 1 ). Subsequent exfoliative cytology of ascites had negative findings. The patient’s poor condition precluded diagnostic laparoscopy. Consequently, we decided to conduct EUS-TTN biopsy for pathological diagnosis. Under EUS guidance, the peritoneum was visualized as a frond-like hyperechoic structure with diffuse thickening contrasted against the anechoic background of the ascites. Utilizing a 19-G fine-needle aspiration (FNA) needle via a transrectal route, the thickened peritoneum was punctured. After the needle tip was positioned into the rectouterine pouch, the stylet was removed and a micro-forceps (disposable micro-forceps; UShare Medical, China) was introduced through the needle to collect peritoneal tissue samples from the rectouterine pouch ( Video 1 ). Three biopsies were taken using the micro-forceps, without adverse events ( Fig. 2 ). Immunohistochemistry identified malignant adenocarcinoma ( Fig. 3 ). The patient underwent chemotherapy with the outcome being a favorable recovery.

**Fig. 1:**
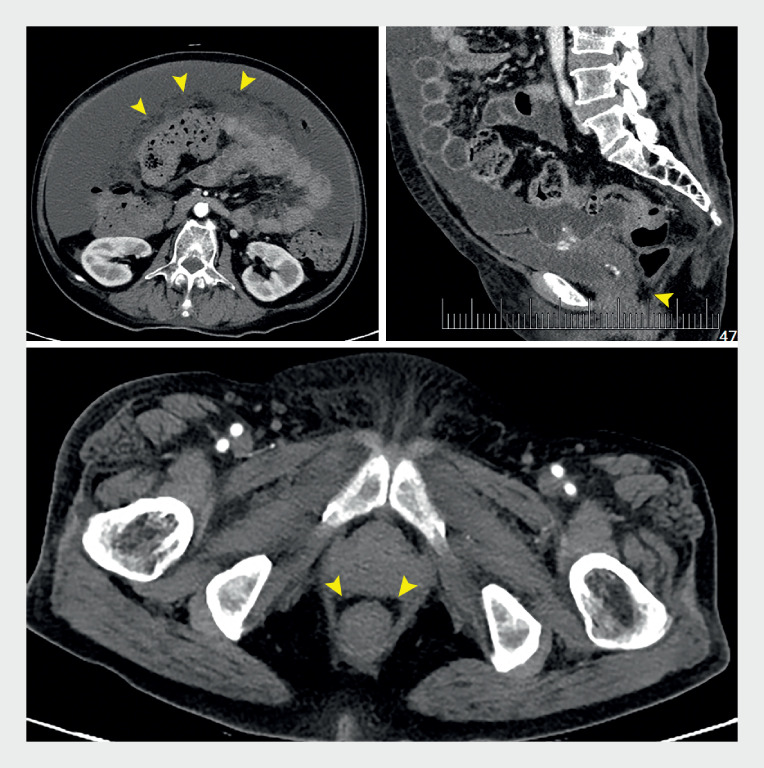
Computed tomography (CT) images; no space-occupying lesions were detected. **a** Diffuse, irregular, sheet-like peritoneal (arrowheads, axial plane). **b, c** Peritoneal thickening in the rectouterine pouch (arrowheads, sagittal and axial planes).

Endoscopic ultrasound-guided through-the-needle biopsy of peritoneal adenocarcinoma.Video 1

**Fig. 2:**
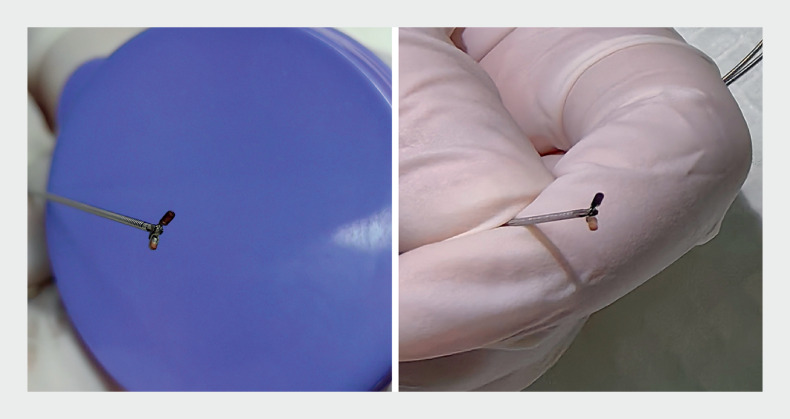
Micro-forceps held by the operator, showing biopsy samples.

**Fig. 3:**
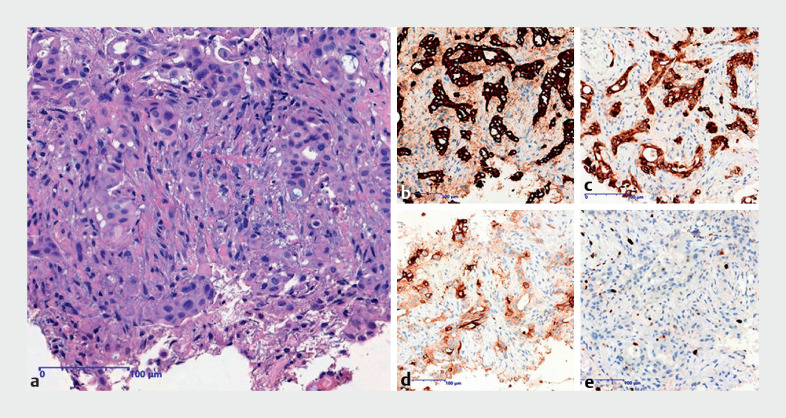
Histopathology of the peritoneal tissue samples. **a** Hematoxylin and eosin staining showed atypical tumor cells (magnification ×200). **b–e** Immunohistochemical staining was positive for AE1/3, EMA, CEA, Ki-67(magnification × 200), respectively.

EUS-TTN biopsy offers a valuable alternative for peritoneal tissue acquisition, especially for patients who are unable to tolerate surgical interventions. The transrectal route is preferred as it allows direct access to peritoneal areas where sedimentation and metastasis are most likely to occur under the influence of gravity.

Endoscopy_UCTN_Code_TTT_1AS_2AZ

## Notes

Endoscopy E-Videos https://eref.thieme.de/e-videos
*E-Videos* is an open access online section of the journal *Endoscopy* , reporting on interesting cases and new techniques in gastroenterological endoscopy. All papers include a high-quality video and are published with a Creative Commons CC-BY license. Endoscopy E-Videos qualify for HINARI discounts and waivers and eligibility is automatically checked during the submission process. We grant 100% waivers to articles whose corresponding authors are based in Group A countries and 50% waivers to those who are based in Group B countries as classified by Research4Life (see: https://www.research4life.org/access/eligibility/ ). This section has its own submission website at https://mc.manuscriptcentral.com/e-videos .
